# ‘It Takes a Village’: Sociocultural Insights From a Qualitative Study on Strategies for Member Engagement and Participation in SMART Recovery

**DOI:** 10.1111/dar.70208

**Published:** 2026-07-07

**Authors:** Wan Jie Tan, Briony Larance, Alison K. Beck, Peter J. Kelly

**Affiliations:** ^1^ School of Psychology University of Wollongong Wollongong Australia

**Keywords:** addiction, cross‐cultural, implementation, self‐help groups, substance use

## Abstract

**Introduction:**

SMART (self‐management and recovery training) recovery is a mutual‐help group that supports and sustains recovery from addiction, including substance use. Meetings are moderated by trained facilitators who manage group dynamics and guide members through a 4‐point program. Nonetheless, sociocultural factors can influence group processes, shaping facilitation style and delivery of SMART Recovery. Our study explored facilitation strategies used in Singapore to foster member engagement and retention.

**Methods:**

In‐depth qualitative interviews with 18 participants (14 members and four facilitators) were analysed using reflexive thematic analysis.

**Results:**

Six themes were generated: (i) collaborative decision‐making to manage inherent hierarchical group dynamics; (ii) using member‐driven topics to find commonality and discuss taboo issues; (iii) emphasising ‘no right or wrong’ alleviates fear of social disapproval and disrupting group harmony; (iv) navigating autonomy within an interdependent group context; (v) encouraging involvement in activities outside of SMART Recovery meetings to strengthen group cohesion; and (vi) aligning facilitation roles with local cultural expectations.

**Discussion and Conclusions:**

Facilitation processes were embedded within broader cultural contexts emphasising interdependence, relational harmony and belonging, collective obligation and avoiding the loss of face. Strategies reflect facilitators' social adaptability, other‐orientation and responsiveness to group dynamics. These promoted key processes such as cohesion, collective identity and ownership, leading to reflective engagement, active participation and retention. Our study reiterates that situating mutual‐help groups within their sociocultural contexts, including the potential coexistence of independent and interdependent views of the self, is essential for understanding and promoting meaningful engagement and participation.

## Key Points

- Members engage and stay when they can understand, relate to and apply the content.
- Flexible and other‐oriented facilitation underpins effective group processes.
- Member‐driven topics make meetings culturally relevant and bridge differences.
- Facilitation strategies work to strengthen cohesion and collective identity.
- Interdependent and independent self‐construals may coexist, shaping group processes.

## 1 Introduction

Mutual‐help groups play a vital role in supporting people in substance use recovery. These groups are often led by members themselves in community‐based settings, making them an integral component of substance use treatment due to their accessibility and affordability [1, 2, 3]. Mutual‐help groups involve shared experiences and collective wisdom, meaningful social connections, emotional support and a sense of belonging and accountability [4, 5, 6]. A variety of mutual‐help groups are available to support and sustain recovery, including 12‐step programs such as Alcoholics Anonymous and Narcotics Anonymous, as well as secular alternatives like SMART (self‐management and recovery training) recovery.

SMART Recovery stands apart from the widely recognised and extensively studied 12‐step programs. It adopts a secular psychological approach grounded in cognitive behavioural therapy, motivational principles and self‐empowerment [7, 8]. Accordingly, it emphasises a more structured format focusing on building skills to support self‐directed change for a range of addictive behaviours [8, 9, 10], while also advocating the appropriate use of professional psychosocial and pharmacological interventions [11]. SMART Recovery groups can vary in size (3–25 participants) and are heterogenous in membership, encompassing individuals with a range of addictive behaviours [12, 13].

Another key distinction lies in facilitation. Unlike 12‐step programs which are entirely peer‐led, SMART Recovery meetings are moderated by trained facilitators who may or may not have lived experience with addiction [8]. Facilitators manage group dynamics and guide members through a 4‐point program: (i) building and maintaining motivation; (ii) coping with urges and cravings; (iii) managing thoughts, feelings and behaviours; and (iv) living a balanced lifestyle [14, 15]. SMART Recovery has expanded significantly worldwide, now offering 3000 SMART meetings across 35 countries in 13 languages [16].

The scientific literature on SMART Recovery has grown in tandem as clinicians and researchers seek to understand its real‐world effectiveness. Current evidence indicates that engagement in SMART Recovery has been associated with increased self‐efficacy and coping skills, and higher rates of continuous abstinence [17, 18]. As evidence continues to grow, it becomes increasingly important to understand the mechanisms through which involvement in SMART Recovery may exert its beneficial effects [7, 11]. While meaningful engagement and the use of coping skills are recognised as key predictors of clinically significant outcomes in mutual‐help groups [19, 20, 21], much of the empirical work has focused on *initiation* (e.g., barriers to treatment) and *outcomes* (e.g., attendance, abstinence, reduction in use). There is scope to further understand the *processes* that sustain engagement and drive participation in SMART Recovery. These processes can interact with individual and environmental factors, including culture [22] and race and ethnicity [23]. As most existing studies have been conducted in predominantly Anglo‐centric regions [10, 24], more work can be done to advance understanding of how these processes, and the strategies that activate them, influence mutual‐help processes in culturally diverse contexts.

The concept of self‐construal (i.e., how individuals define themselves in relation to others) offers a framework in understanding potential cultural differences. An independent self‐construal emphasises autonomy, individuality and self‐expression [25]. This contrasts with the interdependent orientations prevalent in Asian cultures such as Singapore [26], where identity is relational, adaptable and context‐dependent [25, 27] and behaviour is guided by group norms and a sense of duty [28]. With the discourse on the applicability of individualistic recovery frameworks in non‐Western, interdependent cultures [29], adopting a sociocultural perspective may elucidate strategies and processes that foster engagement and participation in mutual‐help groups.

In Singapore, additional sociocultural factors can further shape engagement in SMART Recovery. Norms influenced by Confucian values, such as respect for social roles, duties and responsibilities [30], could influence participation in the interactive, discussion‐based format of SMART Recovery. There is a deference to social hierarchy, defined as an individual's relative influence and agency within a group [31, 32, 33, 34], and individuals in positions of higher status (e.g., leadership, seniority). Additionally, an emphasis on preserving group harmony [35] means that open disagreements may be perceived as socially inappropriate, as public dissent can threaten group harmony or result in a loss of face [36, 37, 38]. The concept of ‘losing face’ refers to the perceived loss of social image or status in the eyes of others when one's actions or words fail to meet or violate social expectations, norms or roles, often leading to embarrassment or shame [39, 40]. In interdependent cultures where group harmony and fulfilment of social duties are highly valued, losing face is particularly significant because it can disrupt interpersonal relationship and threaten group cohesion [41].

Structural features of Singapore's legal system, such as the *Misuse of Drugs Act* and adoption of a zero‐tolerance approach to drug use [42], can also influence engagement. Individuals with drug‐related offences are required to enter treatment through mandated pathways (e.g., Drug Supervision Scheme or Drug Rehabilitation Centre), where recovery decisions are largely determined by professionals and authorities [43]. These structural constraints may limit sense of autonomy and self‐direction [44], which could influence how they engage with SMART Recovery.

Despite the growing global expansion of SMART Recovery, there remains limited research examining its implementation in Singapore and the broader Southeast Asian context [45]. As a multi‐racial, multi‐religious and multi‐cultural society [46], Singapore provides a valuable context for examining how cultural factors could influence engagement in non‐Western contexts. Using a qualitative methodology, we aimed to explore facilitation strategies and their processes that could support meaningful engagement and participation among SMART Recovery members in Singapore. We utilised an existing dataset collected by Tan et al. [47], which examined sociocultural factors influencing members' and facilitators' experiences and perspectives of SMART Recovery. Our present study had a different research aim, extending this work by exploring how facilitators activate processes that sustain engagement and promote participation. Analysis from our study can contribute to culturally sensitive recommendations to enhance support structures and improve participation in SMART Recovery and similar services across the region.

## 2 Methods

### 2.1 Research Design

We adopted a critical realist position to explore strategies and the processes influencing them. Ethical approval was granted by the University of Wollongong Human Research Ethics Committee (protocol number: 2022/303), with letters of support from organisations that facilitate SMART Recovery groups.

### 2.2 Participants

Participants from this study were drawn from a pre‐existing qualitative dataset collected for Tan et al. [47]. Recruited participants were from two recovery centres in Singapore that offered SMART Recovery programs. An explanatory statement was provided verbally to potential participants (members and facilitators). Interested participants were then screened for eligibility based on the following criteria: (i) living in Singapore; (ii) 18 years of age or older; (iii) currently or formerly participating (for members) or facilitating (for facilitators) in SMART Recovery for a substance‐related issue; and (iv) possessing a functional understanding of English (the lingua franca in Singapore). Informed consent was obtained from eligible participants before the interviews, and e‐vouchers for a local supermarket were provided to compensate participants for their time. Recruitment for the original study continued until there was sufficient data to create a rich and complex narrative that fulfilled the study's objectives [48]. The participant group was purposefully selected to reflect diversity in ethnicity, gender, age, educational attainment, income level and length of SMART Recovery attendance. Interviews were conducted by WJT between February and March 2023, who had no prior relationship with the participants and was independent of SMART Recovery.

### 2.3 Dataset Generation

The critical realist position posits that meaning is often hidden and co‐created by the researcher and participant through deep reflections and interpretative dialogue [49]. We utilised two different interview schedules for members and facilitators that followed an in‐depth, semi‐structured format (see Tan et al. [47] and Appendices S1 and S2 for the full interview schedule). Interviews took place in‐person at the recovery centre where SMART Recovery meetings were held (*n* = 17) and online via Zoom (*n* = 1). On average, interviews took 40 min and ranged between 25 and 60 min. All interviews were transcribed by the W.J.T. and included in the analysis. No participants withdrew from the study. Pseudonyms were assigned to maintain participant confidentiality.

### 2.4 Data Analysis

We used reflexive thematic analysis, combining deductive and inductive approaches. The deductive component was guided by existing understanding of the cultural context in Singapore and the study's interview schedules, which informed coding towards anticipated concepts. Concurrently, an inductive approach allowed new themes to be generated directly from participants' perspectives and experiences. According to the critical realist paradigm, reflexivity is crucial because the researchers' social context, experiences, values and assumptions can significantly influence their interpretation of the data [50]. Following the process by Braun and Clarke [51, 52], W.J.T. familiarised herself with the data through transcribing, repeated readings and reflective notes on patterns and nuances. Using Microsoft Excel, initial codes were developed from latent meanings, which were iteratively reviewed and clustered into themes. Themes were refined to reflect facilitation strategies derived from participants' experiences, rather than from a pre‐existing or pre‐determined framework. These were continuously reviewed and discussed within the research team throughout the research process, including near‐final themes, before finalising it in our paper.

### 2.5 Reflexivity and Rigour

W.J.T., a female clinical psychologist trainee from Singapore, holds a Bachelor of Psychological Science (Honours) and is undertaking a PhD in Clinical Psychology and is a bilingual native speaker (English and Mandarin), conducted the interviews and analyses. Other members of the research team comprised individuals with expertise in psychology and substance use interventions and outcomes. P.J.K. is a clinical psychologist and holds a PhD in Clinical Psychology, B.L. holds a PhD, and A.K.B. is a clinical psychologist with a DClinPsych and PhD. All three authors have experience in substance use treatment research and are members of the SMART Recovery Australia Research Advisory Committee. They contributed to the analysis process through iterative discussion and interpretation of findings. This included providing conceptual input, reviewing and refining themes, and offering critical feedback in relation to the theoretical framing and structure of SMART Recovery.

While interviews were conducted in English, it is usual in Singapore for speakers to incorporate words or expressions from other languages within English sentences. This is a common feature of local communication and did not hinder comprehension, as the interviewer was familiar with these expressions. Cultural sensitivities were managed through reflective listening, paraphrasing and clarification to ensure accurate understanding of participants' accounts. Reflexivity and transparency in the research process were maintained through regular discussions with the authorship team to incorporate multiple perspectives and enhance interpretive rigour. A reflective journal was also used to reflect on the researcher's assumptions and positionality, their potential influence on interpretation, and to document the development and refinement of codes and themes [53]. Codes and themes were revisited against the full dataset to ensure that they were consistent and grounded in participants' full accounts, rather than as isolated quotes. The codes and themes were also reviewed by co‐authors who acted as critical friends providing constructive feedback and offering alternative perspectives for data examination [54].

## 3 Results

### 3.1 Participant Characteristics

Eighteen participants (14 SMART Recovery members and four facilitators) were recruited for our study (Table 1). All participants were actively engaged with SMART Recovery at the time of the interview. Participants were aged between 29 and 57 years and were involved with SMART Recovery programs for a duration ranging from 2 months to 5 years (*M* = 2.38 years).

**TABLE 1:** Participant characteristics (*n* = 18).

	Members (*n* = 14)		Facilitators (*n* = 4)		Total (*n* = 18)	
	*n*	% of total	*n*	% of total	*n*	% of total
Ethnicity						
Chinese	9	64.3	3	75.0	12	66.7
Malay	2	14.3	—	—	2	11.1
Indian	1	7.1	1	25.0	2	11.1
Other	2	14.3	—	—	2	11.1
Sikh	1	7.1	—	—	1	5.6
Mixed	1	7.1	—	—	1	5.6
Gender						
Male	10	7.1	2	50.0	12	66.7
Female	3	21.4	2	50.0	5	27.8
Non‐binary/third gender	1	7.1	—	—	1	5.6
Age (*M*, SD)	41.9	9.3	37.3	8.8	40.9	9.1
Education attainment						
Primary school	1	7.1	—	—	1	5.6
Secondary school	4	28.6	—	—	4	22.2
Post‐secondary education	9	64.3	4	100.0	13	72.2
Polytechnic Diploma/Institute of Technical Education/Junior College	4	28.6	—	—	4	22.2
Bachelor's degree	1	7.1	—	—	1	5.6
Postgraduate Diploma/degree	4	28.6	4	100.0	8	44.4
Sources of income^a^						
Employed	6	42.9	4	100.0	10	55.6
Full‐time	2	14.3	4	100.0	6	33.3
Part‐time	3	21.4	—	—	3	16.7
Not specified	1	7.1	—	—	1	5.6
Temporary benefit (e.g., government welfare)	5	35.7	—	—	5	27.8
Dependent on others	1	7.1	—	—	1	5.6
No income	3	21.4	—	—	3	16.7

^a^ Does not sum up to 18 as participants may have multiple sources of income.

### 3.2 Facilitation Strategies That Support Meaningful Engagement and Participation

We generated six themes: (i) collaborative decision‐making to manage inherent hierarchical group dynamics; (ii) using member‐driven topics to find commonality and discuss taboo issues; (iii) emphasising ‘no right or wrong’ alleviates fear of social disapproval and disrupting group harmony; (iv) navigating autonomy within an interdependent group context; (v) encouraging involvement in activities outside of SMART Recovery meetings to strengthen group cohesion; and (vi) aligning facilitation roles with local cultural expectations.

#### 3.2.1 Collaborative Decision‐Making to Manage Inherent Hierarchical Group Dynamics

‘In an Asian context, there is a bit more of a hierarchy when it comes to age’ (Taylor, member, age 30–39). Hierarchies can shape group dynamics and interactions in SMART Recovery. Because SMART Recovery follows an open model, newer members may feel that ‘it's not [their] place to talk about it (substance use)’ (Noah, facilitator, age 30–39) especially in the presence of more experienced peers.

… if you have a huge group of people—disparity, in terms of recovery stage, right. So, a lot of people in late‐stage recovery. Or like, 10 years of recovery, right. They will be having a vastly different conversation to that [sic] one or two individuals inside the room. That's where they (new members) get very intimidated, they won't share. (Noah, facilitator, age 30–39)

Experience with SMART Recovery is not the only factor, as age can also influence perceived authority. As Taylor (member, age 30–39) noted, ‘people who are a bit older, not wanting to get advice or feedback from someone younger’. Similarly, Noah (facilitator, age 30–39) further observed,

There's also the seniority issue. ‘He's a lot older. Is it my place to share? … Everybody is like my father.’ Yeah, age and cultural factors actually play a lot [sic] … I would imagine in a more liberal society, they would have no issues with just sharing. It's my experience, right, it's my unique experience so I will just share. But over here, observation‐wise, Asian youths typically a little bit intimidated. (Noah, facilitator, age 30–39)

Together, these factors highlight how differences in experience with SMART Recovery and age can affect engagement and participation in group discussions. Group size in these meetings can also vary, with larger groups typically comprising ‘a cap of 20 in the room’ (Amelia, facilitator, age 30–39).

To minimise these hierarchical effects, facilitators dispersed authority across members by encouraging and guiding consensus‐based decision‐making, which supported respectful and cohesive group relations. During check‐ins, ‘everyone has an opportunity to … say how they are feeling for the past week, any challenges or any achievements’ (Henry, member, age 40–49). Facilitators then used this information to synthesise recurring themes from these check‐ins to develop discussion topics (e.g., ‘cravings and urges’ [Charles, member, age 40–49]), which the group would collectively ‘agree that this [is what] we want to discuss this week’ (Charles, member, age 40–49). As described by participants, facilitators would ‘list out the topics then people will vote … which topic to talk about’ (Oliver, member, 30–39), or ‘ask us which is more important or more urgent … so everyone can give input’ (Gabriel, member, 50–59).

#### 3.2.2 Using Member‐Driven Topics to Find Commonality and Discuss Taboo Issues

Reflecting on this cultural diversity within SMART Recovery in Singapore, Ava (facilitator, 30–39) noted, ‘we are quite [mixed] here. Like, there's a lot of cultures coming together’. To bridge differences and strengthen cohesion, facilitators emphasised the universality of personal challenges and underlying emotional experiences.

… we usually make sure it's relatable to everyone in the room … we [are] talking about life experiences, we [are] talking about emotions … Because emotions are relatable … We have to be careful in tailoring the conversation such that other people can participate also. (Amelia, facilitator, age 30–39)

Members valued this approach, describing discussions as ‘reflective’ (William, member, age 30–39) and ‘insightful’ (Liam, member, age 40–49). By anchoring discussions in shared emotional experiences and weaving in SMART Recovery tools, members could apply insights to their own circumstances, resulting in richer and more inclusive discussions rather than abstract or ‘textbook’ (Liam, member, age 40–49) ones.

This approach also created a safe space to explore important culturally taboo topics that members may otherwise avoid due to fear of judgement or shame. Recognising shared struggles normalised their experiences and strengthened trust within the group. Nonetheless, for such openness to occur, facilitators themselves needed to be comfortable navigating these taboo topics and discern when an issue is too personal for a group setting. As one participant explained, if facilitators appear uneasy or avoid these conversations, members are more likely to disengage.

If I talk about the sex, and then the facilitator blush or what, I think everyone will just shut down. Everyone will be like ‘Okay, wrong people.’ … No matter the topic, they were very—how to say—not [embarrassed]. Yeah. No matter how taboo the talk is about. Yeah, so it was good. (William, member, age 30–39)

#### 3.2.3 Emphasising ‘No Right or Wrong’ Alleviates Fear of Social Disapproval and Disrupting Group Harmony

One of the main features of SMART Recovery is crosstalk, where members can ‘chip in and share their point of view’ (Liam, member, age 40–49). While this made SMART Recovery meetings more conversational, it can also result in a ‘clash’ due to differing opinions, which can be uncomfortable for members.

People can build upon what other people have said. It can be an opinion, it can be a worldview, you know, that sometimes can lead to differences in opinion, and that might create a clash. (James, facilitator, age 50–59)

Despite the discomfort this approach may cause members, facilitators recognised the value of crosstalk in developing communication skills.

If they can develop that skill and use it in SMART, they can likely use it outside … I learn that just because I have this disagreement with you on some points, doesn't meant that our relationship is broken. We still can sit down and have a meal. (Noah, facilitator, age 30–39)

Nonetheless, the fear of disrupting group harmony or losing face remained a barrier to participation, and the need for ‘safety’ and ‘trust’ was frequently emphasised. Facilitators modelled and encouraged members ‘to be respectful of someone's opinion, no matter whether you agree or disagree’ (Amelia, facilitator, age 30–39). This creates a space where differing perspectives can be shared without fear of embarrassment or judgement.

… we promote safety, alright. We emphasise the fact that, you know, that nobody is getting judged. There is no right or wrong answer … It's like an opportunity for us to share what we feel about the situation, feel about the topic. And not having to feel bad about it. (James, facilitator, age 50–59)

#### 3.2.4 Navigating Autonomy Within an Interdependent Group Context

Participants consistently highlighted the importance of personal agency and ownership over their recovery journey. As one member explained,

All of us are recovering at a different pace and we are recovering at different stages in this journey … That respect that we ourselves are also owners of that journey is important. Rather than taking that away and say ‘Okay, take this. All of you go through 10 weeks of this … All your problems will be solved.’ (Liam, member, age 40–49)

For many members, ownership meant having the autonomy to decide when and how to participate in SMART Recovery meetings. Members appreciated being allowed to ‘ease myself into the meeting’ (Henry, member, age 40–49) without the pressure to speak before feeling ready. Another member reflected,

When I first came here, I was like, I just sat at the corner. For six months … They give me my own time. That's, I think the most helpful to me … If they force me to do anything, I'll probably—maybe I won't come back. Yeah, the initial part is very important. (Ben, member, age 40–49)

The importance of readiness and autonomy are recognised by facilitators, who allowed space for gradual and voluntary engagement. During check‐ins, facilitators paid close attention to whether members preferred to sit‐in and observe, or if they appeared ready to share. Over time, members who initially opted to observe described greater confidence and willingness to participate when they felt emotionally ready and supported by the group.

Despite emphasising individual ownership, members also viewed recovery as a shared process. This may be understood within the meritocratic system in Singapore, emphasising competition and individual ability and effort. Hence, framing recovery as a collective process could help counter this tendency towards social comparison and foster greater openness to receiving support. As one facilitator reflected,

I think in the Singapore context, you know, we really focus on meritocracy. Which means that we are always competitive … whenever we meet somebody, you know, we will be like, already eyeing on how am I better [sic] than that person and where I lose out. And sometimes that can be very damaging because it segregates us … we human beings all don't live alone. Yeah? We rely on each other … we don't realise that we need the help of other. (James, facilitator, age 50–59)

Similarly, Lily (member, age 50–59) reflected, ‘it takes a village, you know, to—yeah, everyone helps each other’ This indicates that for members, autonomy in recovery is exercised alongside an interdependent self‐construal, where personal growth is supported by and contributes to the collective goals of the group.

How we do our recovery may not be same. But somehow, the journey towards that is the same. You know? We are working towards our recovery. You do it your way, I do it my way … It's about connection with the right people and doing our recovery together. (Lily, member, age 50–59)

#### 3.2.5 Encouraging Involvement in Activities Outside of SMART Recovery Meetings to Strengthen Group Cohesion

As SMART Recovery meetings occur only once a week, interactions can feel ‘rather transactional’ (Ethan, member, age 40–49) and may be insufficient for developing closer connections.

For other support groups, you tend to get closer. You exchange numbers, they will look out for you … Whereas for SMART, it's like, after the program is finished, everybody goes their own way. Yeah, and then you do your own things until the next week. (Daniel, member, age 30–39)

Members found that activities beyond SMART Recovery meetings, particularly through shared meals, helped build camaraderie and strengthen bonds. As one member reflected, ‘food is very good for bonding’ (Oliver, member, 30–39). Facilitators likewise encouraged members to join other activities at the recovery centre, recognising that the effectiveness of SMART Recovery depends on ‘what happens before … and after SMART’ (Noah, facilitator, age 30–39).

Going into a meeting only just to talk about recovery and nothing else, and immediately go home right, is not something that will sustain people in the long run. We find that individuals who participate in long‐term … have connections beyond just a SMART session. (Noah, facilitator, age 30–39)

#### 3.2.6 Aligning Facilitation Roles With Local Cultural Expectations

Overall, SMART Recovery facilitators play an important and unique role. Their styles may require adjustment to align with local cultural expectations

Not so much the structure of SMART, but the facilitation of SMART has to be a little bit different … Culturally, I don't actually see many hurdles. (Taylor, member, age 30–39)

Facilitators had to assume various roles and respond flexibly to situational demands and group dynamics. This includes knowing when to be directive and ‘set an agenda’ (Noah, facilitator, age 30–39), ‘allow the bouncing of ideas’ (James, facilitator, age 50–59) or ‘step in and re‐direct the conversation’ (Amelia, facilitator, age 30–39). Rather than adhering rigidly to a prescribed facilitation style or focusing solely on achieving predetermined goals, effective facilitation in this context emphasises on relational responsiveness.

## 4 Discussion

Using in‐depth, semi‐structured qualitative interviews, we explored strategies employed by SMART Recovery facilitators to encourage member engagement and participation. Our study highlights the strategies that support these processes, and the sociocultural factors that shape them. While the core components of SMART Recovery (e.g., 4‐point program) can be utilised within a Singaporean context, careful consideration needs to be given to the facilitation strategies used to support their implementation. This includes flexibility and an other‐oriented perspective that is responsive to group dynamics and members' needs, balancing between directive versus collaborative facilitation approaches.

Cultural factors can shape how hierarchies are perceived and enacted [55]. In Singapore and many other Asian societies, interdependent self‐construals and Confucian values emphasise interpersonal relationships, social harmony and attention to social roles, duties and responsibilities [30]. Respect for authority or seniority is therefore commonly treated with deference [31, 32] and observed in group settings, which may in turn influence participation dynamics in meetings. Within SMART Recovery meetings in Singapore, these cultural factors are taken into consideration through a collaborative setting of agendas and discussion topics based on members' check‐ins. This process allows more equitable participation among members to share their opinions on topics that are considered important, while fostering a sense of ownership over the meeting and affirming their meaningful contribution to the group [56]. This sense of ownership has been linked to greater willingness to participate actively [57], motivation to learn about a topic [58] and integration of diverse perspectives [59]. Furthermore, when individuals perceive themselves as valued, respected and engaged within the group, it further reinforces collective identification and ongoing participation [60, 61]. This approach also provides a safe space for members to discuss important yet culturally sensitive topics, which are often avoided in Asian sociocultural contexts due to concerns about judgement or shame [47, 62, 63].

Many of the strategies focused on group cohesion, characterised by close relationships among members through strong networks, shared identity and purpose, and trust and willingness to cooperate [64]. Group cohesion encourages the internalisation and adoption of values and beliefs as one's own, which is the strongest form of social influence [65]. While shared characteristics may bring individuals together, it is commonality in experiences and meanings that foster comfort and safety [60]. This aligns with research in Singapore that demonstrated the role of social belonging in supporting motivation, identity and engagement in recovery [66]. One way social belonging may be fostered is through the sharing of meals, which many participants in the present study described as helping to strengthen interpersonal bonds. Commensality, also known as the social practice of eating together, can further reinforce group cohesion by cultivating intimacy and collective identity [67, 68] and facilitating the exchange of knowledge and values in many Asian contexts [69, 70]. This is consistent with research showing that individuals with interdependent self‐construals have strong group‐oriented obligations [28] and greater community involvement [71, 72], which extends beyond structured group settings.

For individuals with interdependent self‐construals, particularly in a cultural context where conflict and direct confrontations are avoided to preserve interpersonal relationships and group harmony [35], crosstalk may be uncomfortable. Disagreements are often managed privately, and public dissent may be viewed as challenging collective norms or causing others to lose face, potentially resulting in social disapproval or strained relationships [36, 37, 38]. Despite this cultural incongruity, crosstalk has been recognised by facilitators to develop members' communication skills. This is reflected in Confucian perspectives, which view harmony not as the absence of conflict or blind conformity [30, 35], but as a constructive resolution to achieve balance and mutual understanding [73].

Nonetheless, members in the present study understood recovery as both an individual and shared process. Self‐oriented qualities such as autonomy remained important, occurring in tandem with interdependence. This reflects person‐centred approaches that support individuals to define their own goals and recovery pathways within their circumstances and contexts [74]. By adapting skills to meet one's personal recovery needs, it can foster self‐efficacy and confidence in sustaining recovery [66]. This finding is also consistent with research in Singapore showing that autonomy is a significant predictor of life satisfaction and happiness [75]. The importance of personal ownership can be particularly salient in the Singaporean context, where individuals charged with drug offences often follow mandated treatment pathways under the Drug Supervision Scheme or Drug Rehabilitation Centre [43]. With recovery decisions largely determined by professionals and authorities, compounded by considerable social pressure to conform to a drug‐free narrative [66], individuals may experience limited agency [44]. These structural factors, alongside Singapore's meritocratic system that encourages competition and individual ability and effort [76], may potentially explain why ownership and self‐direction are highly valued by members. Taken together, our findings highlight that independent views of the self can coexist alongside relational, interdependent orientations, offering a deeper understanding of how self‐construals can shape group engagement, participation and cohesion.

Facilitators also play a key role in enhancing engagement. In an interdependent cultural context where sensitivity to relationships (i.e., other‐orientation) and mutual obligations are central to group functioning [25, 28, 77], facilitators adopt flexible roles depending on situational demands, including that of an expert, educator, problem solver, information provider, role model and group leaders [56]. This reflects an attentiveness to members' emotional and interpersonal cues, and adjusting communication accordingly [77]. Consistent with an other‐oriented focus, facilitators were also attentive to members' readiness to participate, allowing space for gradual, voluntary engagement. This aligns with Chung and Bemak's [56] recommendation for working with Asian populations, emphasising that facilitators remain mindful not to be overprotective by allowing members to remain persistently silent, nor excessively confrontational in trying to draw them into participation.

Our study provides foundational, contextual knowledge on facilitation strategies that can inform the development of future interventions in Asian contexts. Nonetheless, our research is potentially limited by its recruitment of current SMART Recovery members, which may introduce a response bias. There may be an over‐ or underrepresentation of certain facilitation strategies, as those who find these strategies beneficial are more likely to continue attending. The small number of facilitators, while reflecting the limited pool of SMART Recovery facilitators in Singapore, may limit the breadth of facilitator‐specific perspectives. Nonetheless, the inclusion of member perspectives supports richness of data, contextualised understanding and multi‐perspectivity appropriate for reflexive thematic analysis. The cross‐sectional design also limits insight into long‐term effects, and future research could clarify whether these strategies contribute to sustained engagement in the long‐term. Strong group identification could also introduce social desirability bias, where participants present overly positive evaluations of the facilitation strategies to protect the group. Despite efforts to encourage open dialogue during the interviews, some participants may feel reluctant to express critical opinions for fear of disrupting group harmony or causing loss of face to themselves or others. The incorporation of triangulation methods in future research, such as other qualitative or quantitative data [78], could better capture self‐reported and observed experiences of members in the group. While this study highlights the role of cultural factors in shaping facilitation styles, participants' perspectives on the broader adaptation of Western/Eurocentric interventions for Asian audiences were not explored. Future research could more directly examine whether adaptation of existing interventions or the development of more localised interventions is preferred. Finally, data collection and analysis were conducted by a single researcher. While this is consistent with reflexive thematic analysis, which positions themes as analytic constructions and emphasises researcher subjectivity, reflexivity and interpretive depth over coding consensus [51, 79, 80], the absence of coding consensus procedures may nevertheless have shaped interpretation of the data [81]. To enhance reflexivity and analytic rigour in the current study, themes were developed through iterative discussions with the authorship team.

## 5 Conclusion

To conclude, the effectiveness of SMART Recovery in Singapore depends not only on its core program components, but also their delivery that enables members to understand, relate to and apply the content. Strategies that emphasise facilitators' flexibility, other‐orientation and responsiveness to group dynamics promote member cohesion and engagement. Many of these facilitation strategies are likely relevant across other Asian and culturally diverse contexts, offering guidance for practitioners seeking to enhance member involvement and retention. In practical terms, this could involve greater emphasis on rapport‐building among members and with facilitators, as well as opportunities for more relational interactions beyond the structured SMART Recovery meetings. It also highlights the importance of facilitators being adaptable and attuned to hierarchical sensitivities, group roles and dynamics, and communication norms. These considerations reiterate the significance of culturally responsive delivery of SMART Recovery in Asian and other non‐Western contexts for it to be acceptable, not by altering its core principles, but by tailoring implementation to align with local cultural norms and expectations. More broadly, these insights highlight the importance of situating mutual‐help groups within their sociocultural contexts, and the need to further our understanding of how group processes influence engagement and participation.

## Author Contributions

**Wan Jie Tan:** conceptualisation, data curation, formal analysis, investigation, methodology, project administration, writing – original draft, writing – review and editing. **Briony Larance:** conceptualisation, methodology, validation, supervision, writing – review and editing. **Alison K. Beck:** validation, writing – review and editing. **Peter J. Kelly:** conceptualisation, methodology, validation, supervision, writing – review and editing.

## Funding

Funding W.J.T. was supported by the International Postgraduate Tuition Awards and University Postgraduate Award at the University of Wollongong while conducting this research. The funding source had no role in study design, analysis and interpretation of data, preparation of manuscript, or any decision to submit the manuscript.

## Conflicts of Interest

B.L., A.K.B. and P.J.K. are members of the SMART Recovery Australia Research Advisory Committee.

## Supporting information


**Appendix S1:** Interview schedule for members.

**Appendix S2:** Interview schedule for facilitators.

## Data Availability

The data that support the findings of this study are available on request from the corresponding author. The data are not publicly available due to privacy or ethical restrictions.
