# Supplementary material for: ‘It Takes a Village’: Sociocultural Insights From a Qualitative Study on Strategies for Member Engagement and Participation in SMART Recovery
Source: Drug Alcohol Rev. 2026 Jul 7;45(5):e70208. doi: 10.1111/dar.70208 (PMC13342422; doi:10.1111/dar.70208)
Supplement: Supplementary file 1 — Appendix S1: Interview schedule for members. [file DAR-45-0-s002.docx]

**Appendix S1. Interview schedule for members**

**Introduction:**

Thank you for taking the time to speak to me for this interview. It will take approximately 45 to 60 minutes to complete, but you can stop at any time you want.

There are no right or wrong answers. I am interested to know your thoughts and opinions about how it feels to be in SMART Recovery. Anything you say will be kept completely confidential, so you are free to mention whatever you want to. If we use any of your words when we write up the study, we will use a pseudonym and make sure that you cannot be identified. I need to highlight that it is a legal requirement to report known cases of illicit substance abuse to the law enforcement agencies, in accordance with the Misuse of Drugs Act (MDA). If you do not want to answer a question, you do not have to, and we can move on.

**Housekeeping:**

- The interview will be audio-recorded to ensure that I do not miss anything important that you have said.
- You can withdraw consent up to one month following the interview.
- Do you have any questions before we start?
- Are you happy to sign the consent form and continue?
- (Wording will be adapted according to participants’ needs. Terminology will be explained if necessary.)

**Contextual question:**

1. As a way of getting started, perhaps you could tell me a little bit about how you knew or heard about SMART Recovery?
   1. Prompt: When did you first attend SMART Recovery?
   2. How long have you been attending SMART Recovery?
   3. What format of SMART Recovery are you using? (e.g., online, face-to-face)
2. What brought you to SMART Recovery?
3. Have you attended any other mutual-aid groups or rehabilitation services in the past?
   1. Is this your first-time using SMART Recovery?

**Questions on perspectives and experiences:**

1. What were your views towards SMART Recovery before you started?
   1. Alternative question: If you had to tell a friend to come to SMART Recovery, what would you say about it?
   2. What were some of these expectations for SMART Recovery?
   3. How have you views towards SMART Recovery changed?
   4. In what ways has the programme met/fell short of your expectations?

Thinking back in time from when you started SMART Recovery until now, can you tell me…

1. What do you like most about SMART Recovery?
   1. Prompt: What is helpful about SMART Recovery?
   2. Prompt: What made it easier for you to attend the SMART Recovery?
   3. Alternative question: From the perspective of a member of SMART Recovery, what are the good things that this program provides?
   4. What do you mean by that?
   5. How did that make you feel?
   6. How important is that to you?
   7. Has what you like changed over time? If so, how and why?
2. What do you not like about SMART Recovery?
   1. Prompt: What do you not find helpful about SMART Recovery?
   2. Prompt: What kind of things has made it difficult for you to attend SMART Recovery?
   3. Alternative question: From the perspective of a member of SMART Recovery, what can be improved about this program?
   4. What do you mean by that?
   5. How did that make you feel?
   6. How important is that to you?
   7. Has what you like changed over time? If so, how and why?

**Questions on culture:**

1. How do you think culture could affect the experience of SMART Recovery?
   1. Individual cultural/religious beliefs and practices
   2. Cultural/religious beliefs and practices of other members in the group
   3. How important is culture to you?
   4. Stigma
2. Has culture affected your experience of SMART Recovery?
   1. If so, in what way?
   2. If not, why did it not affect your experience?
   3. How did the facilitator/host manage this in the group? What actions were taken?
   4. How do you feel about the group?
   5. What do other members think about the group?
3. What changes has been done to make SMART Recovery more appropriate to your cultural values?
   1. What other changes can be done to the program to achieve that?
4. What changes has been done to make SMART Recovery more appropriate for the local cultures in Singapore?
   1. What other changes can be done to the program to achieve that?
5. What do you think about the manual being used in SMART Recovery?
   1. Has it been adapted to suit the local culture?
   2. How do you feel about the questions?
6. What kind of support would you like to have that is not available at SMART Recovery?
7. How does SMART Recovery compare to other recovery services on the cultural aspect?
8. Overall, how would you describe your experience in SMART Recovery?
   1. Prompt: How has it impacted on your social/family life and well-being?

**Ending questions:**

1. Of all the things we discussed, what to you is the most important?
2. Is there anything else you would like to add that we have not covered?
