# Supplementary material for: ‘It Takes a Village’: Sociocultural Insights From a Qualitative Study on Strategies for Member Engagement and Participation in SMART Recovery
Source: Drug Alcohol Rev. 2026 Jul 7;45(5):e70208. doi: 10.1111/dar.70208 (PMC13342422; doi:10.1111/dar.70208)
Supplement: Supplementary file 2 — Appendix S2: Interview schedule for facilitators. [file DAR-45-0-s001.docx]

**Appendix S2. Interview schedule for facilitators**

**Introduction:**

Thank you for taking the time to speak to me for this interview. It will take approximately 45 to 60 minutes to complete, but you can stop at any time you want.

There are no right or wrong answers. I am interested to know your thoughts and opinions about how it feels to facilitate SMART Recovery. Anything you say will be kept completely confidential, so you are free to mention whatever you want to. If we use any of your words when we write up the study, we will use a pseudonym and make sure that you cannot be identified. I need to highlight that it is a legal requirement to report known cases of illicit substance abuse to the law enforcement agencies, in accordance with the Misuse of Drugs Act (MDA). If you do not want to answer a question, you do not have to, and we can move on.

**Housekeeping:**

- The interview will be audio-recorded to ensure that I do not miss anything important that you have said.
- You can withdraw consent up to one month following the interview.
- Do you have any questions before we start?
- Are you happy to sign the consent form and continue?
- (Wording will be adapted according to participants’ needs. Terminology will be explained if necessary.)

**Contextual question:**

1. As a way of getting started, perhaps you could tell me a little bit about how you came to be a facilitator at SMART Recovery?
   1. Prompt: When did you become a facilitator at SMART Recovery?
2. Have you had any experience with other mutual-aid groups or rehabilitation services in the past?

**Questions on perspectives and experiences:**

1. What were your views towards SMART Recovery before you started?
   1. Alternative question: If you had to tell a friend to come to SMART Recovery, what would you say about it?
   2. What were some of these expectations for SMART Recovery?
   3. How have you views towards SMART Recovery changed?
   4. In what ways has the programme met/fell short of your expectations?
2. What training did you receive?
   1. Is there a manual that is used?

Thinking back in time from when you started facilitating SMART Recovery until now, can you tell me…

1. What do you like most about SMART Recovery?
   1. Prompt: What is helpful about SMART Recovery?
   2. Prompt: In your opinion, what made it easier for members to attend the SMART Recovery?
   3. Alternative question: From the perspective of a facilitator of SMART Recovery, what are the good things that this program provides?
   4. What do you mean by that?
   5. How did that make you feel?
   6. How important is that to you?
   7. Has what you like changed over time? If so, how and why?
2. What do you not like about SMART Recovery?
3. Prompt: What do you not find helpful about SMART Recovery?
4. Prompt: In your opinion, what kind of things has made it difficult for members to attend SMART Recovery?
5. Alternative question: From the perspective of a facilitator of SMART Recovery, what can be improved about this program?
6. What do you mean by that?
7. How did that make you feel?
8. How important is that to you?
9. Has what you like changed over time? If so, how and why?

**Questions on culture:**

1. How do you think culture could affect the experience of SMART Recovery?
   1. Individual cultural/religious beliefs and practices
   2. Cultural/religious beliefs and practices of other members in the group
   3. How important is culture to you?
   4. Does the SMART Recovery format takes culture into consideration?
   5. Stigma
2. Has culture affected your experience of SMART Recovery?
   1. If so, in what way?
   2. Has this affected members’ experience of SMART Recovery? If so, in what way?
   3. How is this managed in the group?
3. What changes has been done to make SMART Recovery more appropriate for the local cultures in Singapore?
   1. What other changes can be made?
4. What do you think about the manual being used in SMART Recovery?
   1. Is the manual culturally appropriate? What are some of the things that made it appropriate/not appropriate?
   2. Has it been adapted to suit the local culture? What has been changed to make it more acceptable?
5. What kind of support would you like members to have that is not available at SMART Recovery?
6. How does SMART Recovery compare to other recovery services on the cultural aspect?
7. Overall, how would you describe your experience in SMART Recovery?
8. What do you think is the experience like for members of SMART Recovery?
9. Prompt: How do you think it has impacted on their social/family life and well-being?

**Ending questions:**

1. Of all the things we discussed, what to you is the most important?
2. Is there anything else you would like to add that we have not covered?
